# Management of tobacco dependence in patients with severe mental illness in german-speaking countries: A literature review

**DOI:** 10.1192/j.eurpsy.2021.1541

**Published:** 2021-08-13

**Authors:** D. Gurrea Salas

**Affiliations:** Mental Health Center, Klinikum stuttgart, stuttgart, Germany

**Keywords:** nicotine dependence, Severe mental illness

## Abstract

**Introduction:**

A standardized approach to reduce or decrease the tobacco consumption is not performed. It is being used as a medium to socialize having an educational character on nursing and medical relationship.

**Objectives:**

Current cessation program are thought for patients without relevant cognitive impairments. Evidence about alternative management for this patient subgroup was collected.

**Methods:**

This investigation examined the state of the implementation of nicotine cessation therapy for chronic psychiatric patients in Germany, Austria and Switzerland. German- and English-speaking publications since 2010 were selected. 12 different reviews and control trials were included.

**Results:**

Inpatient experiences from maximum hospital care in Germany have been published in the last 10 years, but mostly by oncologist departments in collaboration with pulmonologists and cardiologists showing a poor interest from psychiatrists, not even for harm reduction strategies in patients with severe mental illness. Therefore, the identification and treatment of nicotine addiction remains very low in patients with mental health conditions. Cognitive and pharmacological interventions are not covered by the German health system.

**Conclusions:**

Latest evidence suggests that more flexible, open-ended, combination approaches of pharmacotherapy and counselling may be more successful. It will hence contribute to redressing the significant health and social inequities experienced by this. population sub-group as a consequence of tobacco smoking.

